# Maintaining transcriptional homeostasis during cell cycle

**DOI:** 10.1080/21541264.2023.2246868

**Published:** 2023-09-01

**Authors:** Lucía Ramos-Alonso, Pierre Chymkowitch

**Affiliations:** Department of Biosciences, Faculty of Mathematics and Natural Sciences, University of Oslo, Oslo, Norway

**Keywords:** Cell cycle, replication, mitosis, transcription memory, chromatin, centromere, Cell Fate

## Abstract

The preservation of gene expression patterns that define cellular identity throughout the cell division cycle is essential to perpetuate cellular lineages. However, the progression of cells through different phases of the cell cycle severely disrupts chromatin accessibility, epigenetic marks, and the recruitment of transcriptional regulators. Notably, chromatin is transiently disassembled during S-phase and undergoes drastic condensation during mitosis, which is a significant challenge to the preservation of gene expression patterns between cell generations. This article delves into the specific gene expression and chromatin regulatory mechanisms that facilitate the preservation of transcriptional identity during replication and mitosis. Furthermore, we emphasize our recent findings revealing the unconventional role of yeast centromeres and mitotic chromosomes in maintaining transcriptional fidelity beyond mitosis.

## Introduction

In metazoans, all cells possess an identical genetic material and a common set of general transcription factors. In theory, this suggests that all genes have the potential to be expressed in any cell type, organ, and temporal context. However, the intricate interplay among specialized transcription factors, chromatin regulators, epigenetic modifications, and cis-regulatory sequences enables cells of distinct tissues to express unique subsets of genes that define and sustain their specific identity and functions. The preservation of these specific gene expression patterns throughout the cell division cycle is essential to perpetuate cellular lineages. Nonetheless, the progression of cells through different phases of the cell cycle significantly disrupts chromatin accessibility, epigenetic marks, and the recruitment of transcriptional regulators. Notably, chromatin in transiently disassembled during S-phase and undergoes drastic condensation during mitosis. Consequently, DNA replication and mitosis present substantial challenges in maintaining the integrity of epigenetic marks, chromatin domains, and gene expression programs that establish cellular identity across cell generations.

During S-phase, cells undergo the process of replicating their entire genomic DNA to ensure the preservation of genetic information after cell division. This replication process involves the progression of the replication fork (RF) along the DNA, which necessitates the disassembly of chromatin. However, this chromatin disassembly poses important challenges in terms of maintaining the integrity of the epigenome and ensuring the propagation of lineage-specific transcription programs. For instance, to package the newly synthesized DNA strands, cells increase the synthesis of histones. However, these newly synthesized histones lack the epigenetic modifications present in the parental histones, just as the replicated DNA strands lack the original methylations. Consequently, the replication process must include the replication of epigenetic marks and DNA methylation patterns on the newly synthesized histones and DNA, respectively, utilizing the parental chromatin and DNA as templates. Furthermore, cells must effectively regulate the imbalance in gene expression resulting from the doubling of the genomic DNA template and resolve collisions between replication and transcription machineries that can lead to genome instability by impeding the progression of the replication fork [1–3].

The replicated DNA and chromatin landscape must then be accurately segregated between the two cells resulting from cell division during M-phase. To do so, early into mitosis, duplicated chromosomes condense thanks to the contraction of chromosome arms and various chromatin modifications. These modifications include the deacetylation and phosphorylation of histones, which promote interactions between nucleosomes [4–8]. As a result, chromosomes become more manageable units for the symmetrical segregation of sister chromatids between daughter cells during cell division. The condensation of mitotic chromatin thus plays a critical role in the accurate transmission of genetic information across successive cellular generations. However, despite the lack of a definitive association between transcription and the higher-order three-dimensional genome architecture, the profound topological and functional changes to chromatin, along with the dissociation of the nuclear membrane, restrict the accessibility of gene regulators to chromatin and trigger significant remodeling of interphase gene expression. This remodeling predominantly involves a global reduction in transcription [9–14]. Consequently, the decrease in gene expression raised the question of how interphase gene expression programs are retained during mitosis and accurately reinstated at the M-G1 transition to ensure the maintenance of cellular identity and functionality. For a long time, the question remained unanswered due to the prevailing notion that mitotic chromatin lacked gene regulatory factors, rendering it transcriptionally inert as a result of chromatin condensation [15–19]. However, this vision of silent mitotic chromatin was partly due to technical limitations. For instance, the presence of general and tissue-specific transcription factors was used as a readout to assess transcription levels via chromatin immunoprecipitation (ChIP). However, the application of formaldehyde crosslinking in ChIP or microscopy caused the translocation of transcription factors to the cytoplasm or failed to capture the swift turnover of transcription regulators on mitotic chromatin [20], which prevented the detection of mitotic gene regulators. In fact, recent studies have provided compelling evidence regarding the preservation of genome accessibility at specific enhancers and promoters during mitosis, despite the loss of long-range intrachromosomal interactions such as topologically associating domains (TADs), enhancer-promoter loops, and enhancer accessibility [7,13,21,22]. This preservation is supported by observations from live-cell imaging, which have demonstrated the co-localization of TFs with mitotic chromatin [23,24]. Furthermore, mass spectrometry analyses of mitotic mammalian cells have revealed the continued presence of numerous transcriptional and chromatin regulators, as well as positive histone marks, on the chromatin throughout mitosis [25,26]. These findings, along with previous studies in *S. cerevisiae* [27], indicated that mitotic chromatin in metazoans is not transcriptionally silent. It became evident that histone marks and gene regulators could i) maintain minimal levels of transcription during mitosis and ii) support mitotic bookmarking, and that both mechanisms may help preserving transcriptional identity throughout M phase (reviewed in [28]).

In this article we describe the gene expression and chromatin regulatory mechanisms that support the perpetuation of transcriptional identity during replication and mitosis. We also emphasize on our recent findings revealing the noncanonical role of yeast centromeres and mitotic chromosomes in safeguarding transcriptional fidelity outside mitosis.

## Perpetuation of transcriptional memory during replication

### Transcription during S-phase

During S-phase, the process of transcription occurs concurrently with DNA replication, giving rise to a potential source of genome instability. This instability arises from the formation of co-transcriptional R-loops, which have the capacity to impede replication forks [29,30]. Several studies have highlighted the role of R-loops in both physiological and pathological contexts (reviewed in [31]). Although most genes remain active during S-phase, cells employ mechanisms to buffer or repress some genes during S-phase to mitigate conflicts between replication and transcription [32]. This has been extensively studied in *Saccharomyces cerevisiae*, where the heterochromatin silencing of the HML and HMR-mating loci is contingent upon progression through S-phase and involves the Sir family of proteins, responsible for removing positive histone marks deposited by Dot1, Sas2, and Rtt109 [33]. Moreover, activation of the Mec1 kinase during S-phase triggers the proteasome-mediated degradation of the RNA polymerase II (RNA pol II) subunit Rpb1, resulting in reduced RNA pol II occupancy on chromatin [34]. Furthermore, under the conditions of replication stress, Mec1 collaborates with the Paf1 and Ino80 complexes to remove RNA pol II and RNA pol III from chromatin [34–36]. Another strategy to prevent genome instability during S-phase involves the exclusion of replication from transcribed regions. For instance, the rDNA locus is flanked by RF-barriers that block replication forks [37]. Additionally, the activity of the helicase Sen1, which is higher during S and G2 phases, contributes to limiting replication-transcription collisions [38,39].

Importantly, numerous genes are specifically transcribed during DNA replication, such as those encoding canonical histones [40,41]. The transcription of histone genes is precisely regulated throughout the cell cycle to prevent an excess or shortage of histone proteins [42]. During S-phase, metazoan cells generate approximately 400 million histone proteins to facilitate the packaging and stabilization of the newly replicated DNA strands. The rate at which histones are synthesized is closely linked to the speed of DNA replication [43]. The initiation of histone gene expression occurs at the G1-S transition through the phosphorylation of the scaffold protein NPAT by Cyclin E/Cdk2, followed by the recruitment of positive transcriptional and chromatin regulators [44,45]. Transcription of histone gene clusters takes place within the histone locus body (HBL), where factors essential for histone gene transcription and pre-mRNA processing, such as FLASH and U7 snRNP, concentrate to facilitate histone production. Note that while the expression of histone genes is secluded to S-phase, the HLB is reassembled immediately after mitosis [45,46].

In budding yeasts, Spt21 and Spt10 enhance the expression of histone genes by promoting histone acetylation in a manner dependent on Cdh1 [47–49]. This increased demand for histone proteins appears to be sustained by an elevated translational capacity, which involves enhanced tRNA synthesis. In budding yeasts, this process relies on the activity of the cyclin-dependent kinase Clb5-Cdk1/Cdc28, which boosts tDNA transcription [50]. In addition to histones, the MET genes are transcribed during S-phase [48,49]. This is likely due to the requirement of S-adenosyl methionine for the biosynthesis of dTTP, which is crucial for DNA replication. In accordance with this, both the mRNA and protein levels of Rnr1 and Rnr3, which are subunits of ribonucleotide-diphosphate reductase responsible for catalyzing the rate-limiting step in dNTP synthesis, reach their peak as cells transition into the S-phase [51]. Another set of genes that is transcribed during S-phase is implicated in the functions associated with the G2/M phases. Yox1 and Yhp1 repressors are responsible for maintaining these genes inactive outside S-phase, but Cdk1/Cdc28 phosphorylates and activates the transcription factor Hcm1 before entry into S-phase, which facilitates the expression of genes involved in various processes such as chromosome organization, spindle dynamics, and budding [52–54]. The expression of the forkhead transcription factor Fkh1 is also observed during S-phase, and it regulates the expression of genes associated with the G2/M phase too [55]. Additionally, Cdk1/Cdc28 promotes the expression of Ndd1, which plays a crucial role in nuclear division during mitosis [56,57]. Interestingly, the expression of centromeric non-coding RNAs is enhanced during the S-phase through the regulation of Cbf1, potentially safeguarding genomic stability [58,59].

### Prevention of gene expression dosage imbalance

Replication progressively duplicates the DNA template accessible to the transcription machinery, resulting in the potential overexpression of early-replicated genes followed by late-replicating ones. Furthermore, replicated chromatin is transiently relaxed by histone acetylation and thus accessible to gene regulators. Several mechanisms are in place to prevent the doubling of gene expression during S-phase. Recent studies in mouse embryonic stem cells (mESCs) have revealed that nascent chromatin remains inaccessible to RNA pol II and undergoes transcriptional silencing for approximately 30 min before returning to normal transcription levels [60]. Accordingly, budding yeast exhibits lower occurrence of RNA pol II on chromatin during S-phase, while the presence of numerous transcription factors (TFs) increases concomitantly with DNA replication [61]. Replicated chromatin thus remains accessible to TFs while RNA pol II is relatively excluded, thereby buffering mRNA synthesis against variations in gene dosage [61,62]. The exclusion of RNA pol II is associated with the H3K56 acetylation of newly-synthesized histones by Rtt109 in yeast [62]. Furthermore, the COMPASS complex (a H3K4 methyltransferase) and its upstream effector PAF1C (a transcription elongation complex) have been proposed to operate downstream of H3K56ac in the buffering mechanism [63]. Consistent with this transcriptional buffering, newly replicated genes exhibit reduced levels of H3K4me3 and an increase in H3K4me2 at their promoters [63]. Another regulator, Elg1, has been implicated in transcriptional buffering during S-phase, as it regulates RNA pol II binding by unloading PCNA during replication [64].

### Chromatin reassembly and perpetuation of histone marks

**Figure 1.**
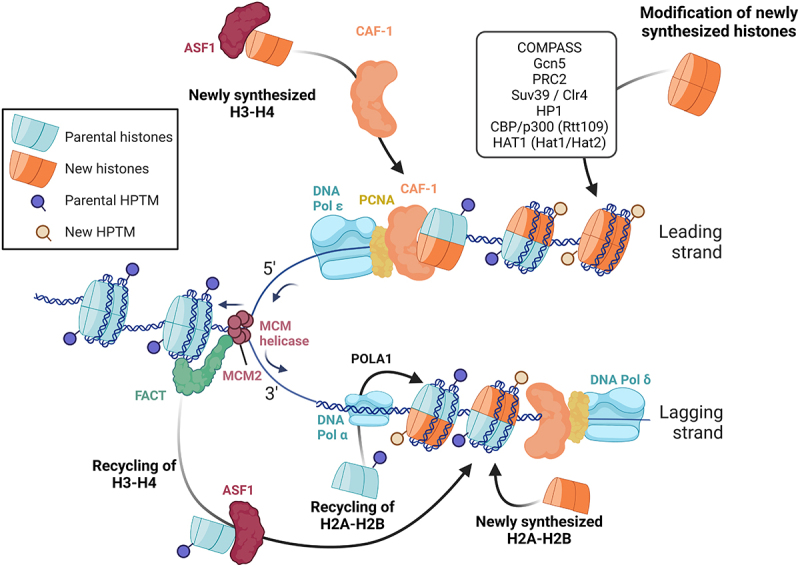
Overview of chromatin replication during S-phase.Note: During replication, parental histones carrying posttranslational modifications (PTMs) are disassembled as the replication fork moves along the DNA. Half of the parental histones and their PTMs are then randomly distributed to each daughter DNA strand. To compensate for the resulting 50% dilution of the pool of histones, newly synthesized histones that lack PTMs are incorporated to daughter strands. To restore a complete epigenetic landscape and preserve transcriptional memory, new histones may be modified before or shortly after their incorporation in the replicated chromatin (for details about the factors and mechanisms involved, see text and references therein).

The faithful repositioning of nucleosomes and the preservation of histone marks during replication play a critical role in maintaining heterochromatin, euchromatin and gene expression memory. Extensive reviews have delved into the process of nucleosome reassembly after replication [3,65,66]. During chromatin replication, the parental histones are distributed between the two replication products, and new histones are incorporated onto the two daughter chromosomes. This ensures that half of the chromatin marks are conserved, which likely helps direct the accurate deposition of marks on the newly synthesized histones. The efficient co-replication recycling of parental histones and the incorporation of new histones onto the daughter strands is achieved thanks to the close interconnection of the replication machinery with nucleosome assembly factors. Notably, the assembly of new H3-H4 dimers is facilitated by the chaperone protein CAF-1, which directly interacts with PCNA, at least on the leading strand [67], while MCM2 serves as a docking site for parental H3-H4 tetramers and may facilitate their recycling by FACT and ASF1 [65]. Although little is known about parental and new H2A-H2B reassembly, recent evidence indicates that the recycling of H2A-H2B is linked to DNA Pol α subunit POLA1, another member of the replisome [68] (Figure 1).

As mentioned above, parental histones undergo dismantling from parental chromatin and each daughter strand randomly receives half of them roughly at their original location, immediately following replication. Remarkably, mass spectrometry experiments have revealed that these histones retain the majority of their posttranslational modifications throughout this process, thus providing substantial evidence in support of epigenetic propagation [68,69]. Nevertheless, it has been observed that repressive marks exhibit a more robust propagation, indicating a preferential preservation of heterochromatin over marks associated with active chromatin during S-phase [70]. Studies conducted in *S. pombe* suggest that this phenomenon can be explained by the larger size of repressed chromatin domains, which facilitates their maintenance compared to smaller regions harboring marks related to active transcription, such as promoters and enhancers [71]. Furthermore, recent findings in mESCs have unveiled the interaction between the chaperone NPM1, the replisome component MCM2, and the Polycomb Repressor Complex 2 (PRC2), which collectively ensures the robust conservation of the repressive mark H3K27me3 during S-phase [72]. The accurate repositioning of nucleosomes by chromatin remodelers, the preservation of nucleosome-free regions at promoters and enhancers and the regulation of transcription factor interaction with these regions during replication have been comprehensively reviewed in [3].

On the other hand, newly synthesized histones lack essential epigenetic marks that necessitate prompt and accurate restoration to maintain chromatin structure and ensure transcription memory (Figure 1). The kinetics of histone mark deposition on nascent histones rely on the nature of the modification itself, wherein positive marks precede negative ones [3]. For instance, H4K5 and H4K12 undergo immediate acetylation during translation in the cytoplasm (by Hat1 and Hat2 in yeast and HAT1 in mammals), and H3K56 is acetylated in the nucleus right before incorporation in daughter strands (by Rtt109 in yeast and CBP/p300 in mammals), which supports genome stability and chromatin assembly [62,73,74]. Moreover, studies conducted in budding yeast have demonstrated that H4K16ac and H3K4me1 rapidly occur on newly synthesized histones following replication, followed by the subsequent appearance of H3K4me3, H3K36me3, and H3K9ac during transcription resumption [75]. Although the precise molecular mechanisms governing the deposition of positive histone marks on nascent histones remain elusive, the aforementioned investigation has revealed that the deposition of H3K4me3 by COMPASS and H3K9ac by Gcn5 occurs more swiftly at TATA-less and highly transcribed genes that promptly regain activity after replication [75,76]. Furthermore, the Spp1 subunit of the COMPASS complex ensures the symmetrical distribution of H3K4me3 on both leading and lagging DNA strands [76]. These findings imply that, at least in the case of H3K4me3, the reactivation of transcription and DNA sequences govern the dynamics of histone modifications on newly synthesized daughter strands, aligning with previous studies reviewed in [77]. Given that H3K4me3 acts as a docking site for TFIID, it is plausible that a positive feedback loop is established to bolster robust gene expression reactivation following replication.

The reestablishment of repressive histone modifications, specifically H3K27me and H3K9me, along with the restoration of heterochromatin domains, exhibits a comparatively slower rate when compared to the deposition of positive marks. The underlying mechanisms involved in this process have been extensively studied and involve reader-writer modules that are capable of recognizing and becoming activated by methylated lysines present on parental histones. Consequently, these modules propagate the methylation signal, leading to the reconstitution of heterochromatin domains [78]. Notably, nucleosomes bearing methylated H3K27 are identified by the chromodomain of the reader-writer PRC2, while nucleosomes marked with H3K9 methylation interact with chromodomains present in H3K9me writers such as Suv39 or Clr4. Furthermore, the maintenance of heterochromatin memory involves HP1, which recognizes H3K9me and recruits methyltransferase partners such as Suv39h1/2 and SETDB1 [79]. Recent studies have also suggested that HP1 contributes to heterochromatin formation through phase separation and homotypic interactions, potentially playing a role in heterochromatin reconstitution and memory during S-phase [10,80]. In certain fungi and mammals, the inheritance of repressive histone methylations additionally involves a crosstalk with methylated DNA, wherein histone and DNA methylation mutually reinforce each other (reviewed in [81]).

### Maintenance of DNA methylation

**Figure 2.**
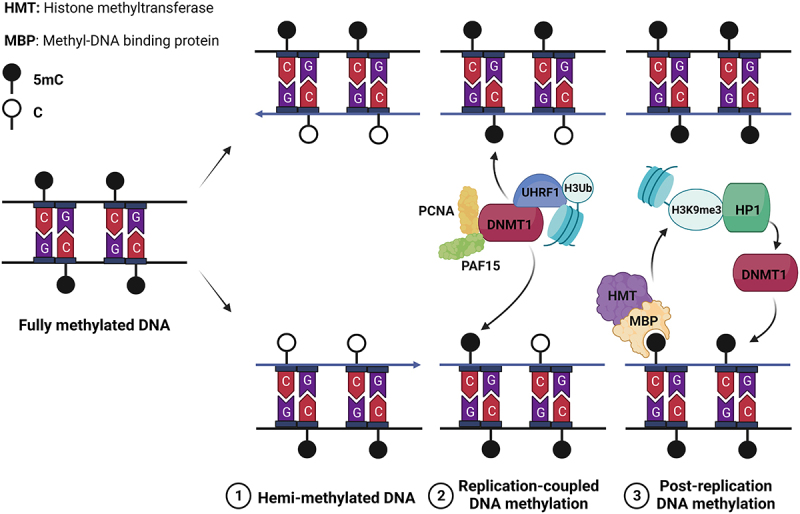
Maintenance of DNA methylation during replication.Note: Daughter DNA strands resulting from replication are hemi-methylated, which is not sufficient to maintain the 5mC landscape and thus chromatin and transcription memory (1). To restore a complete 5mC landscape the maintenance DNA methyltransferase DNMT1 methylates cytosines in two phases of “replication-coupled” (2) and “post-replication” (3) DNA methylation (for details about the factors and mechanisms involved, see text and references therein).

Although DNA methylation is not found in yeasts and some metazoans such as *Drosophila melanogaster* and *Caenorhabditis elegans*, it serves crucial roles in heterochromatin formation, transcriptional repression, embryonic development, and the maintenance of lineage specificity in mammals [82–85]. Consequently, it is imperative to ensure the faithful propagation of specific DNA-methylomes during S-phase to propagate transcriptional programs, chromatin structure, and cellular identity. Nevertheless, akin to histone modifications, a 50% dilution of DNA methylation takes place during S-phase due to the lack of methylation in newly synthesized DNA strands while the parental strands retain their methylation. Consequently, replication generates hemi-methylated double-stranded DNA that necessitates restoration to achieve symmetrical methylation on both strands (Figure 2).

In mammals, the majority of DNA methylation occurs on 5-methyl-cytosines (5mC) within CpG islands. The interplay between DNA methylation and H4K9me3 reinforces the formation of heterochromatin. On the opposite, H3K4me3 and DNA methylation exhibit a mutually exclusive relationship at promoters, leading to the stable repression of gene expression [86]. DNA methylation is achieved by two types of DNA methyltransferases (DNMTs), namely “de novo” and “maintenance” DNMTs. During replication, the “maintenance” DNMT1 methylates hemi-methylated DNA by utilizing the parental methylated DNA strand as a template. The association of DNMT1 with the replication factor PCNA revealed the early coupling of DNA methylation with replication [87]. However, subsequent studies demonstrated that PCNA is dispensable for DNMT1 recruitment, while H3 ubiquitination, UHRF1, PAF15, and H3K9me3 play crucial roles. The activity of DNMT1 can be divided into an initial “replication-coupled” phase involving PAF15 and PCNA, and a subsequent “post-replication” phase dependent on chromatin remodeling and H3K9 methylation [88]. During the later phase, H3K9me3 and DNA methylation reciprocally reinforce each other to reestablish heterochromatin domains. Methyl-DNA binding proteins are responsible for the recognition of 5mC, which in turn recruit histone methyltransferases. On the other hand, HP1 recognizes H3K9me3 and recruits histone methyltransferases and DNMT1 [78,81,89]. This intricate crosstalk between the restoration of histone modifications and DNA methylation is crucial for the faithful perpetuation of transcriptional programs during S-phase.

## Mitotic transcription and bookmarking perpetuate cell identity through mitosis

### Mitotic bookmarking

The observation that a significant number of TFs and numerous histone marks persist on chromatin during mitosis has led to the hypothesis that they function as epigenetic bookmarks, maintaining cell-specific transcriptional identity during the M-phase by marking interphase genes that need to be reactivated upon mitosis exit [12,16,90]. It is important to highlight that, thus far, only a limited set of general transcription factors (GTFs), tissue-specific TFs, and architectural proteins have been demonstrated to remain associated with mitotic chromatin ([91,92] and references therein). This raises questions regarding the molecular mechanisms underlying the selective retention of these gene regulators during mitosis. Currently, two non-exclusive mechanisms have been proposed. First, certain regulators such as TBP, NF-YA, or GAF associate persistently with mitotic chromatin in a sequence-specific manner, thereby potentially preserving the accessibility of chromatin for the transcriptional machinery upon mitotic exit [7,92–94]. Second, gene regulators like ESRRβ and FOXA1 coat chromosomes and dynamically survey mitotic chromatin through both sequence-specific and nonspecific interactions. This establishes a reservoir of TFs in proximity to genes and regulatory sequences, facilitating their mobilization upon mitotic exit [95,96]. Interestingly, the genes with which ESRRβ exhibits a more stable association during mitosis in embryonic stem cells (ESCs) are the ones that are rapidly re-expressed upon mitosis exit, suggesting that the stable interaction of bookmarking TFs with DNA is favored at genes requiring early reactivation during the G1 phase [96]. This example serves as validation for mitotic bookmarking by TFs as one of the mechanisms that perpetuates phenotypes during cell division.

Simultaneously, the experimental alteration of promoter bookmarking often had small effects on interphase transcription, primarily resulting in the delay of proper transcriptome reactivation following cell division without altering it overall. This has raised questions regarding the significance of bookmarking. However, a recent investigation focusing on nuclear receptors NR5A2 and ESRR β shed light on the potential explanation for the lack of observable phenotypes [97]. The study proposed that redundancy among bookmarking factors could account for this phenomenon, wherein multiple gene regulators collaboratively bookmark genes to ensure robust reactivation upon exiting mitosis. Consequently, impairing a single bookmarking factor may not manifest significant phenotypic alterations. Nonetheless, it should be noted that only a fraction of DNA-binding sites occupied by regulators during interphase remain occupied during the M phase, leaving numerous genes unattended by TF bookmarking. Consequently, TF bookmarking alone does not serve as the exclusive mechanism for maintaining transcriptional fidelity during cell division.

An additional mechanism enabling cells to retain and transmit interphase transcription during the M-phase is the bookmarking of histone modifications. These modifications can be associated with both active and repressed gene expression. On one hand, the levels of histone methylation are maintained during the M-phase, encompassing repressive marks such as H3K9me3, H3K27me3, and H3K36me3, as well as active marks like H3K4me2/3 [98]. The latter tend to localize to more punctual genomic regions compared to the former [99]. On the other hand, M-phase is characterized by a general reduction in histone acetylation, including marks such as H3K9ac, H3K14ac, H4K16ac, and H3K18ac [4,98]. However, certain data indicate that H3K27ac remains present at the promoters of housekeeping genes and enhancers of genes that will be reactivated shortly after mitosis exit [100]. Consequently, the inhibition of H3K27 acetylation during mitosis influences the dynamics of transcriptional reactivation during early G1 [101]. These findings indicate that H3K27ac plays a dual role by promoting cell survival during the M-phase while also supporting early G1 transcription. Nevertheless, current data suggest that the active marks retained during mitosis, namely H3K27ac and H3K4me2/3, are in the minority compared to the larger number and wider genomic distribution of repressive marks that persist throughout the M-phase, along with the overall deacetylation of histones [98]. Moreover, repressive marks such as H3K27me3 and H3K9me3 may be deposited and spread in an authentic epigenetic manner through the interaction of writer-reader complexes that are retained on mitotic chromatin, whereas writers of positive marks are depleted [26]. Therefore, cells appear to prioritize the epigenetic memorization of repressed genes and heterochromatin regions during mitosis, rather than the bookmarking of active genes through positive histone modifications [102].

Despite the technical limitations leading to the predominance of correlative studies, current data strongly suggest that bookmarking of gene regulators and histone marks facilitates the memory of interphase transcription during the M phase and supports the reactivation of interphase genes in the early G1 phase. Simultaneously, the existence of less condensed regions within mitotic chromatin and the presence of gene regulators and active histone marks, particularly at promoters, provide evidence for the occurrence of modest levels of transcription during mitosis. These findings suggest a potential role for mitotic transcription in sustaining interphase gene expression.

### Mitotic transcription

Mitosis is characterized by a significant decrease in gene expression, which can be attributed to various factors. These include chromosome condensation and loss of enhancers accessibility, the loss of most promoter-enhancer loops (which could be due to the loss of transcription), and the inhibition of the general transcription machinery. Notably, CDK1 plays a crucial role in this process by phosphorylating certain GTFs, leading to a reduction in transcription [13,21,23,26,93,103–105]. Furthermore, during mitosis entry, specific phosphorylations occur on histone H3 at threonine 3 (H3-T3p) by Aspin and at serine 10 (H3-S10p) by Aurora B. These phosphorylation events not only contribute to chromatin condensation but also play a role in a methyl-phospho switch mechanism. This switch prevents numerous proteins that typically recognize the methylation status of H3 at lysine 4 (H3-K4me) and lysine 9 (H3-K9me) from accessing mitotic chromatin [106]. Notably, H3K4me3 is maintained during mitosis; however, the presence of H3-T3p hinders its recognition by TFIID-TAF3 at gene promoters. As a result, transcriptional initiation is impeded, while maintaining the bookmarking of these regions [107,108]. It is important to note that studies investigating the methyl-phospho switch have primarily relied on *in vitro* experiments and crosslinking-based methodologies. Further investigations are needed to validate these findings under more physiological conditions, employing techniques such as live cell imaging.

However, numerous promoters remain accessible and exhibit low levels of mitotic transcription in yeast and metazoans [14,27,109]. This mitotic RNA synthesis was assessed using metabolic pulse-labeling of nascent RNA in cells arrested with nocodazole [14]. Notably, a remarkable 25% of genes expressed during interphase were also expressed during mitosis, albeit at significantly reduced levels of expression. Additionally, 5% of these mitotic transcripts demonstrated greater expression in M-phase than during interphase, while 3% of mitotic genes remained silent during interphase. These findings not only indicate the preservation of minimal levels of transcription during mitosis but also establish the distinct nature of the mitotic transcriptome compared to that of interphase. Furthermore, it has been observed that mitotic transcription predominantly relies on promoters rather than enhancers, which aligns with the previous discovery that chromatin condensation restricts enhancer accessibility but not that of promoters [14,21,23]. If the purpose of maintaining low levels of mitotic transcription is to transmit cellular identity through cell division, one would anticipate the expression of tissue-specific genes during mitosis and/or their prompt reactivation to interphase levels following mitotic exit. However, genes transcribed during mitosis primarily support fundamental cellular functions, and the transcripts that rapidly achieve high expression levels upon exiting mitosis also pertain to basic cellular functions [14]. In accordance with a recent study employing single-cell ATAC-sequencing to map accessible regions of mitotic chromatin discovered that these regions are marked by NF-YA, which maintains their accessibility and facilitates the rapid reactivation of housekeeping genes upon mitotic exit [7]. Other mechanisms involving GTF, TBP and TFIID, as well as Topoisomerase I, have been demonstrated to sustain promoter accessibility during the M phase and contribute to mitotic transcription or gene reactivation during early G1 (reviewed in [28]).

The recent emergence of advanced techniques such as live cell imaging, Halo-tagging, and mass spectrometry has allowed for the identification of RNA pol II, GTFs, and various gene and chromatin regulators within mitotic chromatin [23–26,93,110]. Subsequent investigations have revealed the presence of an active form of RNA pol II, characterized by phosphorylation on Rpb1-CTD-Ser2 (CTD-S2p), in mitotic fractions. Moreover, chemical inhibition of the CTD-S2 kinase Cdk9 has been shown to impact mitotic transcription [109,110]. These findings of transcriptionally engaged RNA pol II in mitotic chromatin validated the existence of mitotic transcription. Additionally, inhibition of CDK9 has been observed to induce mitotic delays, implying a dependency on Pol II activity for proper mitotic progression, although the precise mechanism remains elusive [110]. The delay may potentially be associated with the localization of RNA pol II at peri-centromeric regions, where it facilitates non-coding transcription to ensure centromere maintenance [111,112]. Moreover, Pol II has been found to interact with Shugoshin, a protein that supports kinetochore function and chromosome segregation [113,114]. Collectively, these investigations suggest that in addition to its roles in mitotic memory and housekeeping functions, mitotic transcription contributes to the progression of mitosis by facilitating the maintenance of centromere and kinetochore integrity.

Limited knowledge exists regarding the phosphorylation of CTD at serine 5 (CTD-S5p) during mitosis. The primary kinase responsible for this phosphorylation, TFIIH-Cdk7, becomes inactive during mitosis, resulting in a decrease in gene expression throughout cell division [115]. However, it is crucial to maintain CTD-S5p at genes expressed during mitosis as it plays a vital role in mRNA capping and promoter escape. Alternative mitotic-specific CTD kinases, such as yeast Cdc15, might fulfill this requirement [116]. Previous studies have demonstrated that human CDC2 and yeast Cdk1/Cdc28 can phosphorylate CTD-S5, establishing a direct connection between transcription and cell cycle regulation [117–119]. It has been proposed that during early G1, Cdk1 collaborates with TFIIH-Cdk7 to phosphorylate CTD-S5, thereby enhancing the expression of housekeeping genes to provide the necessary resources for supporting cell growth [118]. These findings align with current perspectives on mitotic transcription, which postulate that it facilitates the formation of daughter cells. The phosphorylation of CTD-S5 by Cdk1 was recently verified and shown to also support SBF-target genes and cell cycle entry in budding yeast [120]. It would be interesting to further investigate the contribution of Cdk1 to the regulation of RNA polymerase II during M-phase and post-mitotic transcriptional reactivation. Similarly, our understanding of Pol II phosphorylation during mitosis remains limited. Is there a specific CTD code for mitosis? How are CTD phosphorylations selectively removed from silent genes and preserved at mitotic genes in the absence of specific transcription factors and coactivators that regulate pre-initiation complexes? The application of modern techniques, such as cut & run and live cell imaging, which do not involve crosslinking, will undoubtedly shed light on these mechanisms and enhance our comprehension of the occurrence of GTFs, transcription factors (TFs), and histone post-translational modifications (PTMs) during the M-phase.

Another question is how mitotic condensation of the chromatin spares numerous promoters that remain accessible to the transcription machinery. One plausible explanation is that GTFs and RNA Pol II, which persist at promoters to sustain low levels of mRNA synthesis, establish a microenvironment in which their presence and activity facilitate chromatin accessibility throughout M-phase. This proposition has been put forth in the context of interphase gene regulation, wherein GTFs, TFs, co-factors, non-coding RNA (ncRNA), and mRNA synthesis may sustain DNA accessibility at active enhancers and promoters through mechanisms such as phase separation or physical competition with nucleosomes for DNA occupancy [121–124]. Consequently, during M-phase, limited transcriptional activity can elicit local positive feedback, thereby preserving chromatin accessibility at promoters and facilitating mitotic memory and transcriptional reactivation when exiting mitosis. The involvement of RNA Pol II activity, as well as the presence of TBP and H3K27ac on mitotic chromatin, have also been associated with the reestablishment of promoter-enhancer loops and higher-order chromatin structures upon mitosis exit [101,105].

On one hand, accessible promoters drive enhancer-less gene expression during mitosis, resembling the rudimentary transcription units found in yeasts, which also lack enhancers. On the other hand, mitotic transcription plays a crucial role in creating microenvironments of open chromatin at promoters, facilitating the post-mitotic reactivation of interphase transcription and maintaining genome architecture. Nevertheless, the specific upstream signals responsible for maintaining selected gene regulators and epigenetic marks on distinct regions of mitotic chromatin have yet to be elucidated. In the following section, we focus on a newly identified mechanism of mitotic chromosome condensation and on the effect of condensation on interphase transcription.

## Could mitotic condensation of chromosomes support interphase transcription fidelity?

### Centromeres initiate mitotic condensation of chromosomes

**Figure 3.**
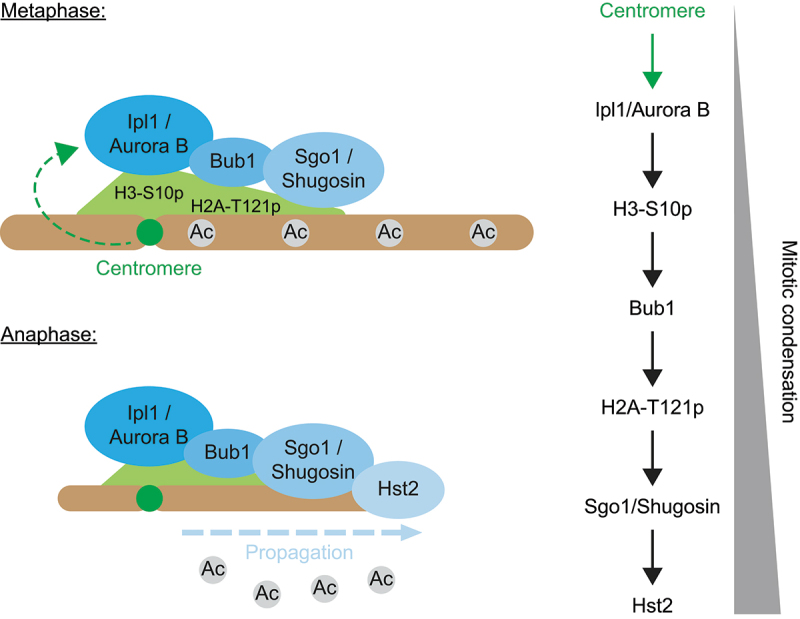
Centromeres instruct mitotic chromosome condensation in budding yeast.Note: Microscopy, ChIP-sequencing and yeast genetics revealed that during metaphase the centromere recruits the activity of Ipl1/AuroraB to the pericentromeric region, which phosphorylates H3 on Ser 10 (H3-S10p). Then, Bub1 is recruited and phosphorylates H2A on T121 (H2A-T121p) to promote the recruitment of Sgo1/Shugosin. During anaphase, Hst2 deacetylates histones and propagates condensation from the pericentromeric region to chromosome arms. In this mechanism the centromere of a chromosome instructs condensation exclusively in *cis*, i.e. without affecting other chromosomes [8].

Until recently, the prevailing understanding of the mechanisms governing chromosome condensation during mitosis focused on the diffusion of signals responsible for chromatin compaction across cell nuclei. However, this conceptual framework has been challenged by recent findings in *S. cerevisiae*, which have revealed an unconventional function of centromeres [8]. The primary role of centromeres has been recognized as providing an anchoring point for kinetochores and microtubules during cell division, thereby ensuring the accurate segregation of condensed mitotic chromosomes. Although this role is conserved across eukaryotes, the DNA sequences of centromeres exhibit no evolutionary conservation. Notably, in higher eukaryotes, centromeres are characterized by epigenetic demarcation and exhibit sequence and positional versatility. In *S. cerevisiae*, however, the centromere consists of a relatively short ~ 140 base pair “point centromere” [125]. This unique feature has facilitated the design of a “centromere excision assay,” wherein the centromere of yeast chromosome IV (*CEN4*) was flanked by LoxP sites, enabling the precise removal of *CEN4* through the expression of a Cre recombinase fused to an estradiol-binding domain [126]. By utilizing this assay, it has been demonstrated that the centromere acts upstream of the kinases Aurora B and Bub1 during mitotic condensation, with Aurora B activity being confined to the peri-centromeric region. Subsequently, Sgo1/Shugoshin and the sirtuin Hst2 contribute to the propagation of the condensation signal onto chromosome arms in *cis* (Figure 3). Thus, at least in *S. cerevisiae*, the initiation of mitotic condensation is governed by a single locus, namely the centromere, and condensation subsequently spreads along chromosome arms in a chromosome-autonomous manner [4,8]. In accordance with this, non-chromosomal DNA species such as DNA circles do not undergo condensation during mitosis and the introduction of a centromere into these circles is sufficient to trigger their condensation [8,127]. An example of such non-chromosomal DNAs are rDNA circles (ERCs), which arise from the rDNA through intra-chromosomal homologous recombination in budding yeast [127]. Intriguingly, while the genomic rDNA is transcribed by RNA polymerases I and III, the rDNA loci present on ERCs are transcribed by RNA polymerase II, which is responsible for mRNA synthesis [128]. Furthermore, the introduction of a centromere into these DNA circles disrupts the association of the RNA Pol II regulator SAGA and interferes with their transcription during mitosis [127]. These observations suggest that the recruitment of the general transcription machinery to chromatin is influenced by the distinction between chromosomal and non-chromosomal DNA contexts. Moreover, this switch in RNA polymerases is likely associated with the remodeling of chromatin architecture at rDNA loci [8,127,129]. These observations suggested that centromeres and mitotic condensation could play an unforeseen role in regulating the dynamics of RNA pol II, GTFs, epigenetic marks and gene expression during M-phase and perhaps beyond mitosis.

### Mitotic chromatin condensation supports transcriptional homeostasis during interphase

**Figure 4.**
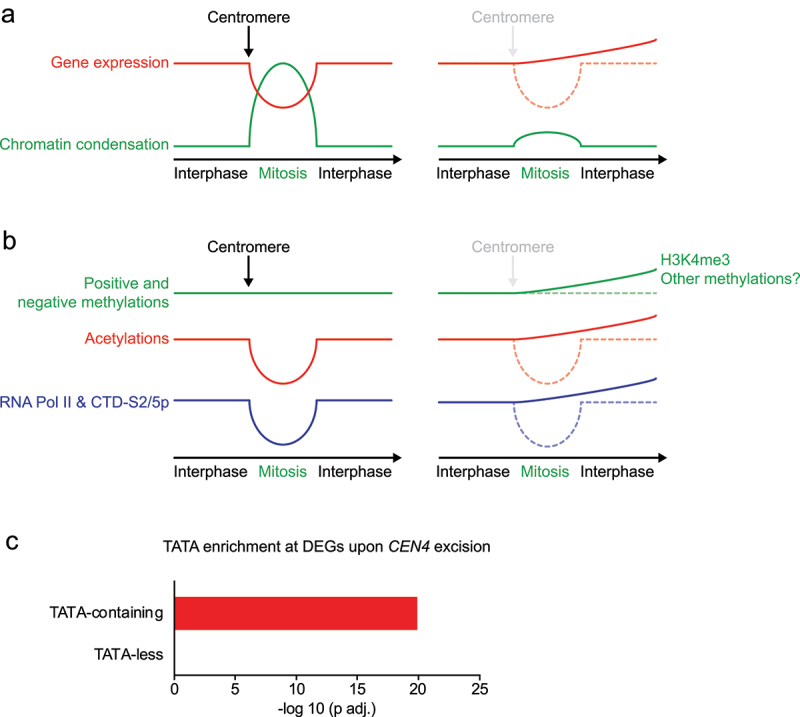
Mitotic chromatin reset chromatin and safeguards interphase transcription fidelity.Note: a) At mitosis entry, centromeres instruct chromosome condensation, which correlate with downregulation of gene expression (left). Centromere inactivation prior to mitosis prevents mitotic condensation, which increases gene expression during M-phase and following interphase (right). b) During M-phase histone methylation remains stable while acetylation decreases. Although minimal levels of mitotic gene expression require the presence of active forms of RNA Pol II, RNA Pol II occurrence during mitosis is lower that during interphase (left). Centromere inactivation prevents mitotic condensation, which correlates with increased occurrence of H3K4me3 and active forms of RNA Pol II. This drives unscheduled transcription, which lasts during the next interphase. c) TATA-containing genes are enriched amongst differentially expressed gene (DEG) upon centromere excision. This analysis was carried out using data published in [130].

In a recent study, we used the centromere excision assay to prevent the mitotic condensation of chromosome IV in budding yeast and investigated the consequences on chromatin and transcriptional regulation [130]. Consistent with previous results [8], ATAC-seq experiments showed a substantial increase in chromatin accessibility on chromosome IV upon excision of *CEN4*, while other chromosomes remained unaffected. Intriguingly, RNA-seq analysis demonstrated that the inactivation of *CEN4*, and thus the absence of mitotic condensation in chromosome IV, induced a comprehensive and selective upregulation of transcription *in cis* (Figure 4a). This observation underscores the repressive impact of mitotic condensation on gene expression, as evidenced by the minimal alterations in transcription when *CEN4* was excised in cells expressing H3-S10D – a mutation that maintains chromosomes in a condensed state in budding yeast [8,130]. Surprisingly, although centromeres mediate condensation *in cis*, transcriptional changes were also observed on other chromosomes. This phenomenon can be attributed to the liberation of genes encoding signal-specific TFs located on chromosome IV from their control mechanisms, resulting in their constitutive expression. Consequently, the uncontrolled expression of these TFs induced the activation of their target genes on other chromosomes. For instance, the chromosome IV gene *GAL3* was expressed even in the absence of galactose, which in turn triggered the expression of Gal3-target genes located on other chromosomes. These findings strongly suggest that the significance of regulating chromatin accessibility, as well as mitotic condensation, may be underestimated in the context of adaptive activation and repression of interphase genes.

Accordingly, the unscheduled transcription of chromosome IV genes correlated with an increase of H4K12ac and H4K16ac occurrence, which is consistent with the function of the sirtuin Hst2 during mitotic condensation [4] (Figures 3 and 4b). The presence of H3K4me3 was also enhanced, suggesting that in the absence of condensation, promoters spontaneously acquire positive methylation, which may subsequently recruit the transcription machinery (Figure 4b). Consistently, the excision of *CEN4* correlated with an increased occurrence of RNA pol II (phosphorylated on CTD-S2 and -S5) on chromosome IV (Figure 4b). These data, along with the limited impact of mitotic condensation on gene expression during M-phase in budding yeast [27], indicate that the inactivation of *CEN4* could affect gene expression beyond mitosis. To investigate this further, cells were arrested before M-phase, and the effect of *CEN4* excision on gene expression was assessed after mitotic exit. Remarkably, the prevention of mitotic condensation had a pronounced, progressive, and persistent impact on transcriptional activation during the subsequent interphase, resulting in sustained hypertranscription of genes located on chromosome IV [130] (Figure 4a,b). Consequently, our findings suggest that the condensation of chromosomes during mitosis prevents the excessive and spontaneous recruitment of the transcription machinery to the chromatin and plays a crucial role in maintaining the fidelity of transcriptional reactivation upon mitotic exit. Additionally, our results suggest that a single locus, the centromere, contributes to the preservation of transcriptional fidelity throughout mitosis and interphase in budding yeast.

## Conclusion and prospects

**Figure 5.**
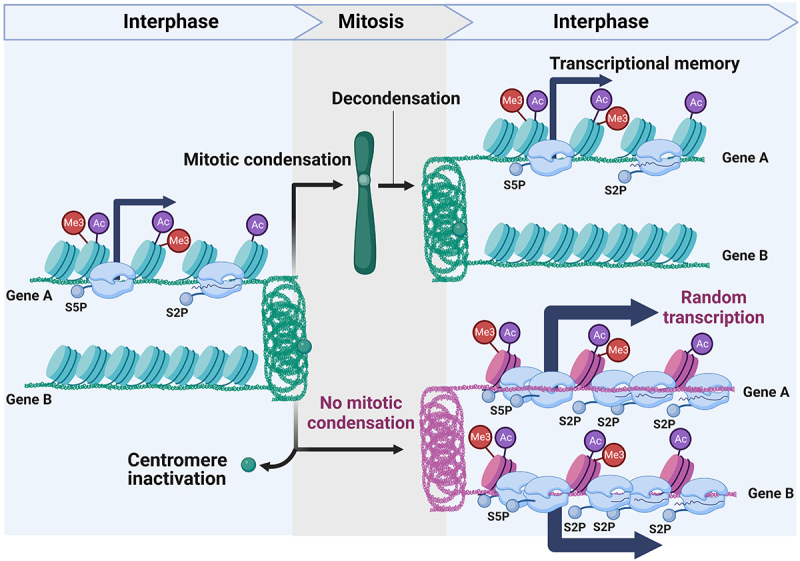
Mitotic condensation of chromosomes safeguards transcriptional homeostasis during interphase.Note: Interphase genes A and B are expressed and repressed, respectively. In normal condition, cells enter mitosis and chromosomes condense, which supports transcriptional memory during the next interphase. On the opposite, preventing chromosome condensation during M-phase precludes chromatin reset, which triggers erratic gene expression during the next interphase [130].

The X shape of mitotic chromosomes has been described in the 19^th^ century. However, our understanding of the structural organization of mitotic chromatin and its impact on chromatin-related processes, such as transcription, continues to expand as we gather more data. The condensation of chromosomes is a multistep process that occurs in a synchronized and controlled fashion. In the case of budding yeast, this condensation process is licensed by the centromere [8] (Figure 3). Our recent study pointed at a novel role of centromeres and mitotic condensation in the regulation of gene expression beyond the M-phase [130] (Figure 5). However, numerous aspects remain unresolved and need further investigation.

Specifically, it remains to be explored whether centromeres exclusively contribute to transcriptional regulation through the spreading of histone deacetylation or if they facilitate additional regulatory mechanisms such as histone methylation or chromatin remodeling [4,8,90,91,93,131]. Additionally, beyond observing an increased occurrence of H4K12/16ac and H3K4me3, we did not look into the mechanistic aspects of how epigenetic marks are deposited and readers recruited upon centromere excision. Regarding histone acetylation, it is tempting to propose that Hst2, which becomes mobilized downstream of the centromere during mitotic condensation [4,8], also plays a role in gene expression reduction during the M-phase. However, other HDACs might be involved, and HATs could be recruited upon centromere inactivation. As for methylation, it is plausible that the COMPASS/Set1 complex is responsible for the increased H3K4me3 levels subsequent to centromere excision, especially considering the role of the mammalian counterpart of COMPASS, the Mixed Lineage Leukemia (MLL) complex, in transcriptional reactivation following mitosis [132]. Thus, we hypothesize that centromeres indirectly suppress the activity of COMPASS upon mitotic entry to prevent unscheduled transcription. On the other hand, we did not explore whether centromeres regulate negative methylation events or the interplay between mitotic phosphorylation of chromatin and the regulation of histone methylation readers. Notably, centromeres recruit Aurora B and facilitate H3-S10 phosphorylation in budding yeast [8], which could hinder the binding of H3-K9me readers through a methyl-phospho switch, thereby limiting transcription [133]. Similarly, a potential connection between the centromere and Haspin-mediated phosphorylation of H3T3, and hence the recognition of H3-K4me3 by TFIID, cannot be discounted [133]. Nevertheless, our findings suggest that centromeres and mitotic condensation in budding yeast contribute to the maintenance of the mitotic histone code, which prepares the chromatin for interphase by promoting histone deacetylation and restricting the occurrence of H3K4me3.

The observed upregulation of H3K4me3 at promoters subsequent to centromere excision suggests that the recruitment of TFIID, and consequently the formation of the pre-initiation complex (PIC), is under the regulation of mitotic condensation. Nevertheless, during mitosis, only a limited number of promoters remain active, as demonstrated by previous studies [27,109]. Thus, the question arises: how does mitotic condensation selectively influence certain promoters while leaving others unaffected? This phenomenon must involve mechanisms other than epigenetic markers such as DNA sequences. Notably, we observed a significant enrichment of differentially expressed genes (DEGs) containing TATA boxes following centromere excision [130,134] (Figure 4c). This finding suggests a specific role for TBP, TFIID, or SAGA (an activator of TBP) in mediating the impact of the centromere on specific genes. This hypothesis aligns with the known involvement of TBP and TFIID in promoter bookmarking [12,135] and the role of the centromere in regulating SAGA’s interaction with ERCs during mitosis [127]. Given that the transcription of ERCs is dependent on TBP, one could postulate that the influence of the centromere on GTFs involves the presence of a TATA box at *CEN4*-regulated genes, which is in line with recent studies that highlighted unexpected roles of TBP/TFIID and SAGA in transcription beyond initially discovered functions [135,136]. Further investigations are needed to explore the link between the centromere, mitotic condensation, TFIID/TBP, SAGA, and the assembly of the pre-initiation complex.

Beyond promoter specificity, an intriguing question is how a single locus affects the expression of genes located anywhere on chromosome arms and not only at the pericentromeric region [130]. This phenomenon of long-range regulation could potentially involve a mechanism wherein the regulatory signal originating from the centromere is propagated, similar to the process observed during chromosome condensation [8]. This mechanism might involve factors that scan the chromatin in a chromosome-autonomous manner, recognizing DNA elements such as the TATA box or epigenetic marks, which has been proposed to contribute to the maintenance of transcriptional fidelity throughout mitosis [137]. Alternatively, since centromeres have been proposed to be a driving force to modulate chromatin architecture, it would be interesting to assess chromatin interactions before and after centromere excision to investigate potential long-range physical interactions between the centromere and genes affect by chromatin condensation [13,138–141].

Interestingly, the data obtained in budding yeast suggest that mitotic chromosome condensation resets the chromatin to avoid a phenomenon that resembles hyper-transcription in mammals [142]. Hypertranscription, driven by oncogenic factors, leads to an excessive recruitment of positive chromatin regulators and general transcription factors. Consequently, it induces replication stress due to collisions between the transcriptional machinery and the replication fork. A question arises regarding the involvement of centromeres in mammalian mitotic condensation and transcriptional homeostasis. However, this remains a challenging question to address as metazoan centromeres are epigenetically demarcated and their on-demand inactivation presents difficulties. The development of novel tools would facilitate investigations into whether centromeres participate in selective inactivation of enhancers and specific promoters during mitosis, as well as whether mitotic condensation effectively prevents unscheduled gene expression beyond the mitotic phase. Notably, our findings may be relevant to asymmetrically dividing cells such as stem cells. In these cells, resetting gene expression programs is vital for supporting pluripotency, cellular identity, and the determination of cell fate [14,90,91,143–145].

Collectively, a multitude of preceding observations and our own research indicate that centromeres and chromosome condensation play pivotal roles in determining cell fate, influencing the aging process, modulating immune responses, and most recently, maintaining transcriptional homeostasis beyond mitosis [113,127,130,146]. A comprehensive exploration of these uncanonical functions of centromeres and mitotic chromatin could reveal fundamental cellular mechanisms and perhaps contribute to our understanding of the pathogenesis of human diseases associated with dysfunctional centromeres [147,148].

## References

[1] Groth A, Rocha W, Verreault A, et al. Chromatin challenges during DNA replication and repair. Cell. 2007;128(4):721–733. doi: 10.1016/j.cell.2007.01.030 17320509

[2] Belotserkovskii BP, Tornaletti S, D’Souza AD, et al. R-loop generation during transcription: formation, processing and cellular outcomes. DNA Repair (Amst). 2018;71:69–81. doi:10.1016/j.dnarep.2018.08.009 30190235 PMC6340742

[3] Stewart-Morgan KR, Petryk N, Groth A. Chromatin replication and epigenetic cell memory. Nat Cell Biol. 2020;22(4):361–371. doi: 10.1038/s41556-020-0487-y 32231312

[4] Wilkins BJ, Rall NA, Ostwal Y, et al. A cascade of histone modifications induces chromatin condensation in mitosis. Science. 2014;343(6166):77–80. doi: 10.1126/science.1244508 24385627

[5] Antonin W, Neumann H. Chromosome condensation and decondensation during mitosis. Curr Opin Cell Biol. 2016;40:15–22. doi: 10.1016/j.ceb.2016.01.013 26895139

[6] Kruitwagen T, Denoth-Lippuner A, Wilkins BJ, et al. Axial contraction and short-range compaction of chromatin synergistically promote mitotic chromosome condensation. Elife. 2015;4:e1039. doi:10.7554/eLife.10396 26615018 PMC4755758

[7] Yu Q, Liu X, Fang J, et al. Dynamics and regulation of mitotic chromatin accessibility bookmarking at single-cell resolution. Sci Adv. 2023;9(4):eadd2175. doi: 10.1126/sciadv.add2175 36696508 PMC9876548

[8] Kruitwagen T, Chymkowitch P, Denoth-Lippuner A, et al. Centromeres license the mitotic condensation of yeast chromosome arms. Cell. 2018;175(3):780–795 e15. doi: 10.1016/j.cell.2018.09.012 30318142 PMC6197839

[9] Rowley MJ, Corces VG. Organizational principles of 3D genome architecture. Nat Rev Genet. 2018;19(12):789–800. doi: 10.1038/s41576-018-0060-8 30367165 PMC6312108

[10] Misteli T. The self-organizing genome: principles of genome architecture and function. Cell. 2020;183(1):28–45. doi: 10.1016/j.cell.2020.09.014 32976797 PMC7541718

[11] Ma Y, Kanakousaki K, Buttitta L. How the cell cycle impacts chromatin architecture and influences cell fate. Front Genet. 2015;6:19. doi: 10.3389/fgene.2015.00019 25691891 PMC4315090

[12] Raccaud M, Suter DM. Transcription factor retention on mitotic chromosomes: regulatory mechanisms and impact on cell fate decisions. FEBS Lett. 2018;592(6):878–887. doi: 10.1002/1873-3468.12828 28862742

[13] Naumova N, Imakaev M, Fudenberg G, et al. Organization of the mitotic chromosome. Science. 2013;342(6161):948–953. doi: 10.1126/science.1236083 24200812 PMC4040465

[14] Palozola KC, Donahue G, Liu H, et al. Mitotic transcription and waves of gene reactivation during mitotic exit. Science. 2017;358(6359):119–122. doi: 10.1126/science.aal4671 28912132 PMC5727891

[15] Prescott DM, Bender MA. Synthesis of RNA and protein during mitosis in mammalian tissue culture cells. Vol. 26. Exp Cell Res; 1962. pp. 260–268. 10.1016/0014-4827(62)90176-3 14488623

[16] Kadauke S, Blobel GA. Mitotic bookmarking by transcription factors. Epigenet Chromatin. 2013;6(1):6. doi: 10.1186/1756-8935-6-6 PMC3621617 23547918

[17] Martinez-Balbas MA, Dey A, Rabindran SK, et al. Displacement of sequence-specific transcription factors from mitotic chromatin. Cell. 1995;83(1):29–38. doi: 10.1016/0092-8674(95)90231-7 7553870

[18] Parsons GG, Spencer CA. Mitotic repression of RNA polymerase II transcription is accompanied by release of transcription elongation complexes. Mol Cell Biol. 1997;17(10):5791–5802. doi: 10.1128/MCB.17.10.5791 9315637 PMC232427

[19] Egli D, Birkhoff G, Eggan K. Mediators of reprogramming: transcription factors and transitions through mitosis. Nat Rev Mol Cell Biol. 2008;9(7):505–516. doi: 10.1038/nrm2439 18568039 PMC7250051

[20] Lerner J, Bagattin A, Verdeguer F, et al. Human mutations affect the epigenetic/bookmarking function of HNF1B. Nucleic Acids Res. 2016;44(17):8097–8111. doi: 10.1093/nar/gkw467 27229139 PMC5041451

[21] Hsiung CC, Morrissey CS, Udugama M, et al. Genome accessibility is widely preserved and locally modulated during mitosis. Genome Res. 2015;25(2):213–225. doi: 10.1101/gr.180646.114 25373146 PMC4315295

[22] Coux RX, Owens NDL, Navarro P. Chromatin accessibility and transcription factor binding through the perspective of mitosis. Transcription. 2020;11(5):236–240. doi: 10.1080/21541264.2020.1825907 33054514 PMC7714440

[23] Teves SS, An L, Hansen AS, et al. A dynamic mode of mitotic bookmarking by transcription factors. Elife. 2016;5: doi: 10.7554/eLife.22280 PMC5156526 27855781

[24] Raccaud M, Friman ET, Alber AB, et al. Mitotic chromosome binding predicts transcription factor properties in interphase. Nat Commun. 2019;10(1):487. doi: 10.1038/s41467-019-08417-5 30700703 PMC6353955

[25] Ohta S, Bukowski-Wills J-C, Sanchez-Pulido L, et al. The protein composition of mitotic chromosomes determined using multiclassifier combinatorial proteomics. Cell. 2010;142(5):810–821. doi: 10.1016/j.cell.2010.07.047 20813266 PMC2982257

[26] Ginno PA, Burger L, Seebacher J, et al. Cell cycle-resolved chromatin proteomics reveals the extent of mitotic preservation of the genomic regulatory landscape. Nat Commun. 2018;9(1):4048. doi: 10.1038/s41467-018-06007-5 30279501 PMC6168604

[27] Granovskaia MV, Jensen LJ, Ritchie ME, et al. High-resolution transcription atlas of the mitotic cell cycle in budding yeast. Genome Biol. 2010;11(3):R24. doi: 10.1186/gb-2010-11-3-r24 20193063 PMC2864564

[28] Ito K, Zaret KS. Maintaining transcriptional specificity through mitosis. Annu Rev Genomics Hum Genet. 2022;23(1):53–71. doi: 10.1146/annurev-genom-121321-094603 35440147 PMC9976632

[29] Garcia-Muse T, Aguilera A. R loops: from physiological to pathological roles. Cell. 2019;179(3):604–618. doi: 10.1016/j.cell.2019.08.055 31607512

[30] Bayona-Feliu A, Aguilera A. The role of chromatin at transcription-replication conflicts as a genome safeguard. Biochem Soc Trans. 2021;49(6):2727–2736. doi: 10.1042/BST20210691 34821364

[31] Petermann E, Lan L, Zou L. Sources, resolution and physiological relevance of R-loops and RNA–DNA hybrids. Nat Rev Mol Cell Biol. 2022;23(8):521–540. doi: 10.1038/s41580-022-00474-x 35459910

[32] Gaillard H, Aguilera A. Transcription as a threat to genome integrity. Annu Rev Biochem. 2016;85(1):291–317. doi: 10.1146/annurev-biochem-060815-014908 27023844

[33] Goodnight D, Rine J. S-phase-independent silencing establishment in Saccharomyces cerevisiae. Elife. 2020;9: doi: 10.7554/eLife.58910 PMC7398696 32687055

[34] Hurst V, Challa K, Jonas F, et al. A regulatory phosphorylation site on Mec1 controls chromatin occupancy of RNA polymerases during replication stress. EMBO J. 2021;40(21):e108439. doi: 10.15252/embj.2021108439 34569643 PMC8561635

[35] Poli J, Gerhold C-B, Tosi A, et al. Mec1, INO80, and the PAF1 complex cooperate to limit transcription replication conflicts through RNAPII removal during replication stress. Genes Dev. 2016;30(3):337–354. doi: 10.1101/gad.273813.115 26798134 PMC4743062

[36] Bhalla P, Shukla A, Vernekar DV, et al. Yeast PAF1 complex counters the pol III accumulation and replication stress on the tRNA genes. Sci Rep. 2019;9(1):12892. doi: 10.1038/s41598-019-49316-5 31501524 PMC6733944

[37] Egidi A, Di Felice F, Camilloni G. Saccharomyces cerevisiae rDNA as super-hub: the region where replication, transcription and recombination meet. Cell Mol Life Sci. 2020;77(23):4787–4798. doi: 10.1007/s00018-020-03562-3 32476055 PMC11104796

[38] Mischo HE, Chun Y, Harlen KM, et al. Cell-cycle modulation of transcription termination factor Sen1. Mol Cell. 2018;70(2):312–326 e7. doi: 10.1016/j.molcel.2018.03.010 29656924 PMC5919780

[39] Alzu A, Bermejo R, Begnis M, et al. Senataxin associates with replication forks to protect fork integrity across RNA-polymerase-II-transcribed genes. Cell. 2012;151(4):835–846. doi: 10.1016/j.cell.2012.09.041 23141540 PMC3494831

[40] Hereford LM, Osley MA, Ludwig II JR, et al. Cell-cycle regulation of yeast histone mRNA. Cell. 1981;24(2):367–375. doi: 10.1016/0092-8674(81)90326-3 7016339

[41] Hereford L, Bromley S, Osley MA. Periodic transcription of yeast histone genes. Cell. 1982;30(1):305–310. doi: 10.1016/0092-8674(82)90036-8 6751560

[42] Kurat CF, Recht J, Radovani E, et al. Regulation of histone gene transcription in yeast. Cell Mol Life Sci. 2014;71(4):599–613. doi: 10.1007/s00018-013-1443-9 23974242 PMC11113579

[43] Groth A, Corpet A, Cook AJL, et al. Regulation of replication fork progression through histone supply and demand. Science. 2007;318(5858):1928–1931. doi: 10.1126/science.1148992 18096807

[44] Duronio RJ, Marzluff WF. Coordinating cell cycle-regulated histone gene expression through assembly and function of the histone locus body. RNA Biol. 2017;14(6):726–738. doi: 10.1080/15476286.2016.1265198 28059623 PMC5519241

[45] Armstrong C, Passanisi VJ, Ashraf HM, et al. Cyclin E/CDK2 and feedback from soluble histone protein regulate the S phase burst of histone biosynthesis. Cell Rep. 2023;42(7):112768. doi: 10.1016/j.celrep.2023.112768 37428633 PMC10440735

[46] Hur W, Kemp JP, Tarzia M, et al. CDK-Regulated phase separation Seeded by histone genes ensures precise growth and function of histone locus bodies. Dev Cell. 2020;54(3):379–394 e6. doi: 10.1016/j.devcel.2020.06.003 32579968 PMC7423771

[47] Kurat CF, Lambert J-P, Petschnigg J, et al. Cell cycle-regulated oscillator coordinates core histone gene transcription through histone acetylation. Proc Natl Acad Sci U S A. 2014;111(39):14124–14129. doi: 10.1073/pnas.1414024111 25228766 PMC4191790

[48] Cho RJ, Campbell MJ, Winzeler EA, et al. A genome-wide transcriptional analysis of the mitotic cell cycle. Mol Cell. 1998;2(1):65–73. doi: 10.1016/S1097-2765(00)80114-8 9702192

[49] Spellman PT, Sherlock G, Zhang MQ, et al. Comprehensive Identification of cell cycle–regulated genes of the yeast Saccharomyces cerevisiae by microarray hybridization. Mol Biol Cell. 1998;9(12):3273–3297. doi: 10.1091/mbc.9.12.3273 9843569 PMC25624

[50] Herrera MC, Chymkowitch P, Robertson JM, et al. Cdk1 gates cell cycle-dependent tRNA synthesis by regulating RNA polymerase III activity. Nucleic Acids Res. 2018;46(22):11698–11711. doi: 10.1093/nar/gky846 30247619 PMC6294503

[51] Blank HM, Papoulas O, Maitra N, et al. Abundances of transcripts, proteins, and metabolites in the cell cycle of budding yeast reveal coordinate control of lipid metabolism. Mol Biol Cell. 2020;31(10):1069–1084. doi: 10.1091/mbc.E19-12-0708 32129706 PMC7346729

[52] Landry BD, Mapa CE, Arsenault HE, et al. Regulation of a transcription factor network by Cdk1 coordinates late cell cycle gene expression. EMBO J. 2014;33(9):1044–1060. doi: 10.1002/embj.201386877 24714560 PMC4193936

[53] Pramila T, Wu W, Miles S, et al. The forkhead transcription factor Hcm1 regulates chromosome segregation genes and fills the S-phase gap in the transcriptional circuitry of the cell cycle. Genes Dev. 2006;20(16):2266–2278. doi: 10.1101/gad.1450606 16912276 PMC1553209

[54] Conti MM, Li R, Narváez Ramos MA, et al. Phosphosite scanning reveals a complex phosphorylation code underlying CDK-dependent activation of Hcm1. Nat Commun. 2023;14(1):310. doi: 10.1038/s41467-023-36035-9 36658165 PMC9852432

[55] Simon I, Barnett J, Hannett N, et al. Serial regulation of transcriptional regulators in the yeast cell cycle. Cell. 2001;106(6):697–708. doi: 10.1016/S0092-8674(01)00494-9 11572776

[56] Koranda M, Schleiffer A, Endler L, et al. Forkhead-like transcription factors recruit Ndd1 to the chromatin of G2/M-specific promoters. Nature. 2000;406(6791):94–98. doi: 10.1038/35017589 10894549

[57] Loy CJ, Lydall D, Surana U. NDD1, a high-dosage suppressor of cdc28-1N, is essential for expression of a subset of late-S-phase-specific genes in Saccharomyces cerevisiae. Mol Cell Biol. 1999;19(5):3312–3327. doi: 10.1128/MCB.19.5.3312 10207056 PMC84125

[58] Ling YH, Yuen KWY. Point centromere activity requires an optimal level of centromeric noncoding RNA. Proc Natl Acad Sci U S A. 2019;116(13):6270–6279. doi: 10.1073/pnas.1821384116 30850541 PMC6442628

[59] Hedouin S, Logsdon GA, Underwood JG, et al. A transcriptional roadblock protects yeast centromeres. Nucleic Acids Res. 2022;50(14):7801–7815. doi: 10.1093/nar/gkac117 35253883 PMC9371891

[60] Stewart-Morgan KR, Reverón-Gómez N, Groth A. Transcription restart establishes chromatin accessibility after DNA replication. Mol Cell. 2019;75(2):284–297.e6. doi: 10.1016/j.molcel.2019.04.033 31126739

[61] Bar-Ziv R, Brodsky S, Chapal M, et al. Transcription factor binding to replicated DNA. Cell Rep. 2020;30(12):3989–3995.e4. doi: 10.1016/j.celrep.2020.02.114 32209462

[62] Voichek Y, Bar-Ziv R, Barkai N. Expression homeostasis during DNA replication. Science. 2016;351(6277):1087–1090. doi: 10.1126/science.aad1162 26941319

[63] Voichek Y, Mittelman K, Gordon Y, et al. Epigenetic control of expression homeostasis during replication is stabilized by the replication checkpoint. Mol Cell. 2018;70(6):1121–1133.e9. doi: 10.1016/j.molcel.2018.05.015 29910110

[64] Huang JH, Liao Y-R, Lin T-C, et al. iTARGEX analysis of yeast deletome reveals novel regulators of transcriptional buffering in S phase and protein turnover. Nucleic Acids Res. 2021;49(13):7318–7329. doi: 10.1093/nar/gkab555 34197604 PMC8287957

[65] Hammond CM, Strømme CB, Huang H, et al. Histone chaperone networks shaping chromatin function. Nat Rev Mol Cell Biol. 2017;18(3):141–158. doi: 10.1038/nrm.2016.159 28053344 PMC5319910

[66] Grover P, Asa JS, Campos EI. H3–H4 histone chaperone pathways. Ann Rev Genet. 2018;52(1):109–130. doi: 10.1146/annurev-genet-120417-031547 30183406

[67] Rouillon C, Eckhardt BV, Kollenstart L, et al. CAF-1 deposits newly synthesized histones during DNA replication using distinct mechanisms on the leading and lagging strands. Nucleic Acids Res. 2023;51(8):3770–3792. doi: 10.1093/nar/gkad171 36942484 PMC10164577

[68] Flury V, Reverón-Gómez N, Alcaraz N, et al. Recycling of modified H2A-H2B provides short-term memory of chromatin states. Cell. 2023;186(5):1050–1065 e19. doi: 10.1016/j.cell.2023.01.007 36750094 PMC9994263

[69] Alabert C, Barth TK, Reverón-Gómez N, et al. Two distinct modes for propagation of histone PTMs across the cell cycle. Genes Dev. 2015;29(6):585–590. doi: 10.1101/gad.256354.114 25792596 PMC4378191

[70] Escobar TM, Oksuz O, Saldaña-Meyer R, et al. Active and repressed chromatin domains exhibit distinct nucleosome segregation during DNA replication. Cell. 2019;179(4):953–963 e11. doi: 10.1016/j.cell.2019.10.009 31675501 PMC6917041

[71] Dodd IB, Micheelsen MA, Sneppen K, et al. Theoretical analysis of epigenetic cell memory by nucleosome modification. Cell. 2007;129(4):813–822. doi: 10.1016/j.cell.2007.02.053 17512413

[72] Escobar TM, Yu J-R, Liu S, et al. Inheritance of repressed chromatin domains during S phase requires the histone chaperone NPM1. Sci Adv. 2022;8(17):eabm3945. doi: 10.1126/sciadv.abm3945 35476441 PMC9045712

[73] Nagarajan P, Ge Z, Sirbu B, et al. Histone acetyl transferase 1 is essential for mammalian development, genome stability, and the processing of newly synthesized histones H3 and H4. PLoS Genet. 2013;9(6):e1003518. doi: 10.1371/journal.pgen.1003518 23754951 PMC3675013

[74] Zhang Y, Zhang Q, Zhang Y, et al. The role of histone modification in DNA replication-coupled nucleosome assembly and cancer. Int J Mol Sci. 2023;24(5):4939. doi: 10.3390/ijms24054939 36902370 PMC10003558

[75] Bar-Ziv R, Voichek Y, Barkai N. Chromatin dynamics during DNA replication. Genome Res. 2016;26(9):1245–1256. doi: 10.1101/gr.201244.115 27225843 PMC5052047

[76] Serra-Cardona A, Duan S, Yu C, et al. H3K4me3 recognition by the COMPASS complex facilitates the restoration of this histone mark following DNA replication. Sci Adv. 2022;8(18):eabm6246. doi: 10.1126/sciadv.abm6246 35544640 PMC9075808

[77] Howe FS, Fischl H, Murray SC, et al. Is H3K4me3 instructive for transcription activation? BioEssays. 2017;39(1):1–12. doi: 10.1002/bies.201600095 28004446

[78] Allshire RC, Madhani HD. Ten Principles of heterochromatin formation and function. Nat Rev Mol Cell Biol. 2018;19(4):229–244. doi: 10.1038/nrm.2017.119 29235574 PMC6822695

[79] Hathaway NA, Bell O, Hodges C, et al. Dynamics and memory of heterochromatin in Living cells. Cell. 2012;149(7):1447–1460. doi: 10.1016/j.cell.2012.03.052 22704655 PMC3422694

[80] Strom AR, Emelyanov AV, Mir M, et al. Phase separation drives heterochromatin domain formation. Nature. 2017;547(7662):241–245. doi: 10.1038/nature22989 28636597 PMC6022742

[81] Du J, Johnson LM, Jacobsen SE, et al. DNA methylation pathways and their crosstalk with histone methylation. Nat Rev Mol Cell Biol. 2015;16(9):519–532. doi: 10.1038/nrm4043 26296162 PMC4672940

[82] Neri F, Rapelli S, Krepelova A, et al. Intragenic DNA methylation prevents spurious transcription initiation. Nature. 2017;543(7643):72–77. doi: 10.1038/nature21373 28225755

[83] Ambrosi C, Manzo M, Baubec T. Dynamics and context-dependent roles of DNA methylation. J Mol Biol. 2017;429(10):1459–1475. doi: 10.1016/j.jmb.2017.02.008 28214512

[84] Dahlet T, Argüeso Lleida A, Al Adhami H, et al. Genome-wide analysis in the mouse embryo reveals the importance of DNA methylation for transcription integrity. Nat Commun. 2020;11(1):3153. doi: 10.1038/s41467-020-16919-w 32561758 PMC7305168

[85] Greenberg MVC, Bourc’his D. The diverse roles of DNA methylation in mammalian development and disease. Nat Rev Mol Cell Biol. 2019;20(10):590–607. doi: 10.1038/s41580-019-0159-6 31399642

[86] Rose NR, Klose RJ. Understanding the relationship between DNA methylation and histone lysine methylation. Biochim Biophys Acta. 2014;1839(12):1362–1372. doi: 10.1016/j.bbagrm.2014.02.007 24560929 PMC4316174

[87] Leonhardt H, Page AW, Weier H-U, et al. A targeting sequence directs DNA methyltransferase to sites of DNA replication in mammalian nuclei. Cell. 1992;71(5):865–873. doi: 10.1016/0092-8674(92)90561-P 1423634

[88] Petryk N, Bultmann S, Bartke T, et al. Staying true to yourself: mechanisms of DNA methylation maintenance in mammals. Nucleic Acids Res. 2020;49(6):3020–3032. doi: 10.1093/nar/gkaa1154 PMC8034647 33300031

[89] Estève PO, Chin HG, Smallwood A, et al. Direct interaction between DNMT1 and G9a coordinates DNA and histone methylation during replication. Genes Dev. 2006;20(22):3089–3103. doi: 10.1101/gad.1463706 17085482 PMC1635145

[90] Liu Y, Pelham-Webb B, Di Giammartino DC, et al. Widespread mitotic bookmarking by histone marks and transcription factors in pluripotent stem cells. Cell Rep. 2017;19(7):1283–1293. doi: 10.1016/j.celrep.2017.04.067 28514649 PMC5495017

[91] Palozola KC, Lerner J, Zaret KS. A changing paradigm of transcriptional memory propagation through mitosis. Nat Rev Mol Cell Biol. 2019;20(1):55–64. doi: 10.1038/s41580-018-0077-z 30420736 PMC6557398

[92] Festuccia N, Gonzalez I, Owens N, et al. Mitotic bookmarking in development and stem cells. Development. 2017;144(20):3633–3645. doi: 10.1242/dev.146522 29042475

[93] Teves SS, An L, Bhargava-Shah A, et al. A stable mode of bookmarking by TBP recruits RNA polymerase II to mitotic chromosomes. Elife. 2018;7: doi: 10.7554/eLife.35621 PMC6037474 29939130

[94] Bellec M, Dufourt J, Hunt G, et al. The control of transcriptional memory by stable mitotic bookmarking. Nat Commun. 2022;13(1):1176. doi: 10.1038/s41467-022-28855-y 35246556 PMC8897465

[95] Caravaca JM, Donahue G, Becker JS, et al. Bookmarking by specific and nonspecific binding of FoxA1 pioneer factor to mitotic chromosomes. Genes Dev. 2013;27(3):251–260. doi: 10.1101/gad.206458.112 23355396 PMC3576511

[96] Festuccia N, Dubois A, Vandormael-Pournin S, et al. Mitotic binding of Esrrb marks key regulatory regions of the pluripotency network. Nat Cell Biol. 2016;18(11):1139–1148. doi: 10.1038/ncb3418 27723719

[97] Chervova A, Molliex A, Baymaz HI, et al. Mitotic bookmarking redundancy by nuclear receptors mediates robust post-mitotic reactivation of the pluripotency network. bioRxiv. 2022;2022.11.28.518105.

[98] Javasky E, Shamir I, Gandhi S, et al. Study of mitotic chromatin supports a model of bookmarking by histone modifications and reveals nucleosome deposition patterns. Genome Res. 2018;28(10):1455–1466. doi: 10.1101/gr.230300.117 30166406 PMC6169886

[99] Valls E, Sanchez-Molina S, Martinez-Balbas MA. Role of histone modifications in marking and activating genes through mitosis. J Biol Chem. 2005;280(52):42592–42600. doi: 10.1074/jbc.M507407200 16199528

[100] Hsiung CC, Bartman CR, Huang P, et al. A hyperactive transcriptional state marks genome reactivation at the mitosis–G1 transition. Genes Dev. 2016;30(12):1423–1439. doi: 10.1101/gad.280859.116 27340175 PMC4926865

[101] Pelham-Webb B, Polyzos A, Wojenski L, et al. H3K27ac bookmarking promotes rapid post-mitotic activation of the pluripotent stem cell program without impacting 3D chromatin reorganization. Mol Cell. 2021;81(8):1732–1748 e8. doi: 10.1016/j.molcel.2021.02.032 33730542 PMC8052294

[102] Reinberg D, Vales LD. Chromatin domains rich in inheritance. Science. 2018;361(6397):33–34. doi: 10.1126/science.aat7871 29976815

[103] Enserink JM, Chymkowitch P. Cell cycle-dependent transcription: the cyclin dependent kinase Cdk1 is a direct regulator of basal transcription machineries. Int J Mol Sci. 2022;23(3):1293. doi: 10.3390/ijms23031293 35163213 PMC8835803

[104] Festuccia N, Owens N, Papadopoulou T, et al. Transcription factor activity and nucleosome organization in mitosis. Genome Res. 2019;29(2):250–260. doi: 10.1101/gr.243048.118 30655337 PMC6360816

[105] Zhang H, Emerson DJ, Gilgenast TG, et al. Chromatin structure dynamics during the mitosis-to-G1 phase transition. Nature. 2019;576(7785):158–162. doi: 10.1038/s41586-019-1778-y 31776509 PMC6895436

[106] de Castro IJ, Gokhan E, Vagnarelli P. Resetting a functional G1 nucleus after mitosis. Chromosoma. 2016;125(4):607–619. doi: 10.1007/s00412-015-0561-6 26728621 PMC5023730

[107] Varier RA, Outchkourov NS, de Graaf P, et al. A phospho/methyl switch at histone H3 regulates TFIID association with mitotic chromosomes. EMBO J. 2010;29(23):3967–3978. doi: 10.1038/emboj.2010.261 20953165 PMC3020634

[108] Gatchalian J, Gallardo CM, Shinsky SA, et al. Chromatin condensation and recruitment of PHD finger proteins to histone H3K4me3 are mutually exclusive. Nucleic Acids Res. 2016;44(13):6102–6112. doi: 10.1093/nar/gkw193 27016734 PMC5291243

[109] Palozola KC, Liu H, Nicetto D, et al. Low-level, global transcription during mitosis and dynamic gene reactivation during mitotic exit. Cold Spring Harb Symp Quant Biol; 2018. 10.1101/sqb.2017.82.034280 29348325

[110] Liang K, Woodfin A, Slaughter B, et al. Mitotic transcriptional activation: clearance of actively engaged pol II via transcriptional elongation control in mitosis. Mol Cell. 2015;60(3):435–445. doi: 10.1016/j.molcel.2015.09.021 26527278 PMC5548174

[111] Gent JI, Dawe RK. RNA as a structural and regulatory component of the centromere. Ann Rev Genet. 2012;46(1):443–453. doi: 10.1146/annurev-genet-110711-155419 22974300

[112] Saffery R, Sumer H, Hassan S, et al. Transcription within a functional human centromere. Mol Cell. 2003;12(2):509–516. 14536089 10.1016/s1097-2765(03)00279-x

[113] Liu H, Qu Q, Warrington R, et al. Mitotic transcription Installs Sgo1 at centromeres to coordinate chromosome segregation. Mol Cell. 2015;59(3):426–436. 26190260 10.1016/j.molcel.2015.06.018

[114] Chan FL, Marshall OJ, Saffery R, et al. Active transcription and essential role of RNA polymerase II at the centromere during mitosis. Proc Natl Acad Sci U S A. 2012;109(6):1979–1984. doi: 10.1073/pnas.1108705109 22308327 PMC3277563

[115] Akoulitchev S, Reinberg D. The molecular mechanism of mitotic inhibition of TFIIH is mediated by phosphorylation of CDK7. Genes Dev. 1998;12(22):3541–3550. 9832506 10.1101/gad.12.22.3541 PMC317239

[116] Singh AK, Rastogi S, Shukla H, et al. Cdc15 phosphorylates the C-terminal domain of RNA polymerase II for transcription during mitosis. J Biol Chem. 2017;292(13):5507–5518. doi: 10.1074/jbc.M116.761056 28202544 PMC5392693

[117] Chymkowitch P, Eldholm V, Lorenz S, et al. Cdc28 kinase activity regulates the basal transcription machinery at a subset of genes. Proc Natl Acad Sci U S A. 2012;109(26):10450–10455. doi: 10.1073/pnas.1200067109 22689984 PMC3387082

[118] Chymkowitch P, Enserink JM. The cell cycle rallies the transcription cycle: Cdc28/Cdk1 is a cell cycle-regulated transcriptional CDK. Transcription. 2013;4(1):3–6. 23131667 10.4161/trns.22456 PMC3644040

[119] Zhang J, Corden JL. Identification of phosphorylation sites in the repetitive carboxyl-terminal domain of the mouse RNA polymerase II largest subunit. J Biol Chem. 1991;266(4):2290–2296. doi: 10.1016/S0021-9258(18)52242-0 1899239

[120] Koivomagi M, Swaffer MP, Turner JJ, et al. G(1) cyclin-Cdk promotes cell cycle entry through localized phosphorylation of RNA polymerase II. Science. 2021;374(6565):347–351. 34648313 10.1126/science.aba5186 PMC8608368

[121] Mousavi K, Zare H, Dell'orso S, et al. eRnas promote transcription by establishing chromatin accessibility at defined genomic loci. Mol Cell. 2013;51(5):606–617. 23993744 10.1016/j.molcel.2013.07.022 PMC3786356

[122] Gilchrist DA, Dos Santos G, Fargo DC, et al. Pausing of RNA polymerase II disrupts DNA-specified nucleosome organization to enable precise gene regulation. Cell. 2010;143(4):540–551. doi: 10.1016/j.cell.2010.10.004 21074046 PMC2991113

[123] Hnisz D, Shrinivas K, Young RA, et al. A phase separation model for transcriptional control. Cell. 2017;169(1):13–23. doi: 10.1016/j.cell.2017.02.007 28340338 PMC5432200

[124] Henninger JE, Oksuz O, Shrinivas K, et al. RNA-Mediated feedback control of transcriptional condensates. Cell. 2021;184(1):207–225 e24. doi: 10.1016/j.cell.2020.11.030 33333019 PMC8128340

[125] Marshall OJ, Chueh AC, Wong LH, et al. Neocentromeres: new insights into centromere structure, disease development, and karyotype evolution. Am J Hum Genet. 2008;82(2):261–282. doi: 10.1016/j.ajhg.2007.11.009 18252209 PMC2427194

[126] Warsi TH, Navarro MS, Bachant J, et al. DNA topoisomerase II is a determinant of the tensile properties of yeast centromeric chromatin and the tension checkpoint. Mol Biol Cell. 2008;19(10):4421–4433. doi: 10.1091/mbc.e08-05-0547 18701701 PMC2555933

[127] Denoth-Lippuner A, Krzyzanowski MK, Stober C, et al. Role of SAGA in the asymmetric segregation of DNA circles during yeast ageing. Elife. 2014;3: doi: 10.7554/eLife.03790 PMC4232608 25402830

[128] Conrad-Webb H, Butow RA. A polymerase switch in the synthesis of rRNA in saccharomyces cerevisiae. Mol Cell Biol. 1995;15(5):2420–2428. doi: 10.1128/MCB.15.5.2420 7739526 PMC230471

[129] Vu L, Siddiqi I, Lee B-S, et al. RNA polymerase switch in transcription of yeast rDNA: role of transcription factor UAF (upstream activation factor) in silencing rDNA transcription by RNA polymerase II. Proc Natl Acad Sci U S A. 1999;96(8):4390–4395. doi: 10.1073/pnas.96.8.4390 10200272 PMC16342

[130] Ramos-Alonso L, Holland P, Le Gras S, et al. Mitotic chromosome condensation resets chromatin to safeguard transcriptional homeostasis during interphase. Proc Natl Acad Sci U S A. 2023;120(4):e2210593120. doi: 10.1073/pnas.2210593120 36656860 PMC9942888

[131] Hsu JY, Sun Z-W, Li X, et al. Mitotic phosphorylation of histone H3 is governed by Ipl1/aurora kinase and Glc7/PP1 phosphatase in budding yeast and nematodes. Cell. 2000;102(3):279–291. doi: 10.1016/S0092-8674(00)00034-9 10975519

[132] Blobel GA, Kadauke S, Wang E, et al. A reconfigured pattern of MLL occupancy within mitotic chromatin promotes rapid transcriptional reactivation following mitotic exit. Mol Cell. 2009;36(6):970–983. doi: 10.1016/j.molcel.2009.12.001 20064463 PMC2818742

[133] van Wely KH, Mora Gallardo C, Vann KR, et al. Epigenetic countermarks in mitotic chromosome condensation. Nucleus. 2017;8(2):144–149. doi: 10.1080/19491034.2016.1276144 28045584 PMC5403135

[134] Rhee HS, Pugh BF. Genome-wide structure and organization of eukaryotic pre-initiation complexes. Nature. 2012;483(7389):295–301. doi: 10.1038/nature10799 22258509 PMC3306527

[135] Joo YJ, Ficarro SB, Soares LM, et al. Downstream promoter interactions of TFIID TAFs facilitate transcription reinitiation. Genes Dev. 2017;31(21):2162–2174. doi: 10.1101/gad.306324.117 29203645 PMC5749164

[136] Baptista T, Grünberg S, Minoungou N, et al. SAGA is a general cofactor for RNA polymerase II transcription. Mol Cell. 2017;68(1):130–143 e5. doi: 10.1016/j.molcel.2017.08.016 28918903 PMC5632562

[137] Gonzalez I, Molliex A, Navarro P. Mitotic memories of gene activity. Curr Opin Cell Biol. 2021;69:41–47. doi: 10.1016/j.ceb.2020.12.009 33454629

[138] Cavalli G, Misteli T. Functional implications of genome topology. Nat Struct Mol Biol. 2013;20(3):290–299. doi: 10.1038/nsmb.2474 23463314 PMC6320674

[139] Siersbaek R, Madsen JGS, Javierre BM, et al. Dynamic rewiring of promoter-anchored chromatin loops during adipocyte differentiation. Mol Cell. 2017;66(3):420–435 e5. doi: 10.1016/j.molcel.2017.04.010 28475875

[140] Zhang H, Blobel GA. Genome folding dynamics during the M-to-G1-phase transition. Curr Opin Genet Dev. 2023;80:102036. doi: 10.1016/j.gde.2023.102036 37099832 PMC10280458

[141] Tjong H, Li W, Kalhor R, et al. Population-based 3D genome structure analysis reveals driving forces in spatial genome organization. Proc Natl Acad Sci U S A. 2016;113(12):E1663–72. doi: 10.1073/pnas.1512577113 26951677 PMC4812752

[142] Bowry A, Kelly RDW, Petermann E. Hypertranscription and replication stress in cancer. Trends Cancer. 2021;7(9):863–877. doi: 10.1016/j.trecan.2021.04.006 34052137

[143] Neurohr G, Naegeli A, Titos I, et al. A midzone-based ruler adjusts chromosome compaction to anaphase spindle length. Science. 2011;332(6028):465–468. doi: 10.1126/science.1201578 21393511

[144] Roubinet C, White IJ, Baum B. Asymmetric nuclear division in neural stem cells generates sibling nuclei that differ in size, envelope composition, and chromatin organization. Curr Biol. 2021;31(18):3973–3983 e4. doi: 10.1016/j.cub.2021.06.063 34297912 PMC8491657

[145] Timmers HTM, Verrijzer CP. Mitotic chromosomes: not so silent after all. Dev Cell. 2017;43(2):119–121. doi: 10.1016/j.devcel.2017.10.002 29065303

[146] Tran V, Lim C, Xie J, et al. Asymmetric division of Drosophila male germline stem cell shows asymmetric histone distribution. Science. 2012;338(6107):679–682. doi: 10.1126/science.1226028 23118191 PMC3532436

[147] Barra V, Fachinetti D. The dark side of centromeres: types, causes and consequences of structural abnormalities implicating centromeric DNA. Nat Commun. 2018;9(1):4340. doi: 10.1038/s41467-018-06545-y 30337534 PMC6194107

[148] Smurova K, De Wulf P. Centromere and Pericentromere transcription: roles and regulation … in sickness and in health. Front Genet. 2018;9:674. doi: 10.3389/fgene.2018.00674 30627137 PMC6309819

